# Mind Wandering, Sleep Quality, Affect and Chronotype: An Exploratory Study

**DOI:** 10.1371/journal.pone.0091285

**Published:** 2014-03-07

**Authors:** Richard Carciofo, Feng Du, Nan Song, Kan Zhang

**Affiliations:** 1 Key Laboratory of Behavorial Science, Institute of Psychology, Chinese Academy of Sciences, Beijing, China; 2 College of Humanities and Social Sciences, Graduate University of the Chinese Academy of Sciences, Beijing, China; 3 Training College, Beijing Foreign Studies University, Beijing, China; University College London, United Kingdom

## Abstract

Poor sleep quality impairs cognition, including executive functions and concentration, but there has been little direct research on the relationships between sleep quality and mind wandering or daydreaming. Evening chronotype is associated with poor sleep quality, more mind wandering and more daydreaming; negative affect is also a mutual correlate. This exploratory study investigated how mind wandering and daydreaming are related to different aspects of sleep quality, and whether sleep quality influences the relationships between mind wandering/daydreaming and negative affect, and mind wandering/daydreaming and chronotype. Three surveys (*Ns* = 213; 190; 270) were completed with Chinese adults aged 18–50, including measures of sleep quality, daytime sleepiness, mind wandering, daydreaming, chronotype and affect (positive and negative). Higher frequencies of mind wandering and daydreaming were associated with poorer sleep quality, in particular with poor subjective sleep quality and increased sleep latency, night-time disturbance, daytime dysfunction and daytime sleepiness. Poor sleep quality was found to partially mediate the relationships between daydreaming and negative affect, and mind wandering and negative affect. Additionally, low positive affect and poor sleep quality, in conjunction, fully mediated the relationships between chronotype and mind wandering, and chronotype and daydreaming. The relationships between mind wandering/daydreaming and positive affect were also moderated by chronotype, being weaker in those with a morning preference. Finally, while daytime sleepiness was positively correlated with daydream frequency, it was negatively correlated with a measure of problem-solving daydreams, indicating that more refined distinctions between different forms of daydreaming or mind wandering are warranted. Overall, the evidence is suggestive of a bi-directional relationship between poor sleep quality and mind wandering/daydreaming, which may be important in attempts to deal with sleep problems and improve sleep quality. These findings and further research on this topic may also have implications for definitions and theories of mind wandering and daydreaming.

## Introduction

The concepts of mind wandering and daydreaming are difficult to precisely define and distinguish, encompassing a wide variety of spontaneous and undirected mentation [1], but they are often used to refer to the shifting of attention away from the external environment, and any ongoing task, towards (task-unrelated) internally-focused mentation [2], [3]. This seems to occur frequently for most people, being reported in approximately 30–50% of thought-probe responses in laboratory and field studies (e.g., [4], [5]). Although mind wandering or daydreaming may be productive, by, for example, facilitating problem-solving, planning and creativity [6], they can have deleterious effects on a wide range of task performance, including reading and memory tasks ([2], [6] for reviews).

### Mind Wandering and Sleep Quality

Impaired task performance is also associated with sleep disturbance. Lack of sleep, through acute sleep deprivation or chronic sleep restriction, is associated with impaired cognitive functioning and state instability, including attention lapses, slower reaction times, impaired working memory [7]–[9], and possibly also,“… intrusive daydreaming while engaged in cognitive work” ( [10], p.210). EEG evidence indicates that mind wandering states are similar to those of low alertness [11], and although the relationship between sleep and mind wandering or daydreaming has received little direct investigation, evidence for an association has been noted in many studies (e.g., [4], [12], [13], [14], [15], [16]). For example, Antrobus et al. [12] found that general ratings of drowsiness were associated with ‘daydream-like mentation’ (although changes in drowsiness ratings between conditions were not accompanied by changes in daydreaming), and other laboratory-based (e.g., [14], [15]) and field-based (e.g., [4]) studies have found positive correlations between reports of mind wandering and sleepiness or tiredness. Furthermore, Kunzendorf et al. [13] found a strong negative correlation between self-reported hours of sleep per night and the frequency of daydreaming, and Mikulincer et al. [16] found that task-unrelated thought increased over 72-hours of sleep deprivation (although only in participants rated as high in trait mind wandering).

Other evidence suggests that forms of mind wandering may be a cause of lack of sleep or poor sleep quality. For example, worry and rumination may increase sleep latency [17], [18], which is also related to emotional responses to stress, such as anxiety and depression [19], [20], [21]. Additionally, Ottaviani and Couyoumdjian [22] found that non-ruminative mind wandering during the day (as assessed by field-based thought-sampling) significantly predicted difficulty in getting to sleep that evening.

### Mind Wandering, Sleep Quality and Negative Affect

Many studies show that much mind wandering or daydreaming is associated with negative affect. For example, Giambra and Traynor [23] found significant positive correlations between the frequencies of daydreaming and mind wandering and three different questionnaire measures of depression, while several studies (e.g., [24]) have found that mind wandering is associated with dysphoria. Also, a large-scale thought-sampling study [25] found that mind wandering predicted subsequent negative affect, while an experimental study [26] found evidence for the inverse relationship, as inducing negative mood increased the frequency of mind wandering in a subsequent experimental task. Stawarczyk et al. [27] also found that daydream frequency was correlated with depression and anxiety, but additionally found that mindfulness (in conjunction with a measure of internal versus external encoding), fully mediated the relationship of daydream frequency and a brief measure of psychological distress (i.e., daydream frequency was no longer a significant predictor of distress). Although there are still issues in defining mindfulness [28] it essentially involves attentive awareness of the present moment [29]. It is associated with good quality sleep, well-being, and positive affect [30], and is negatively correlated with daytime sleepiness [31], and with mind wandering and daydreaming [22], [32], [33]. In contrast, sleep deprivation or poor quality sleep are associated with negative affect, anxiety and depression [34]–[36]. Taken together, these findings suggest that any relationships between mind wandering or daydreaming and negative affect, may also involve sleep quality.

### Mind Wandering, Sleep Quality, Negative Affect and Chronotype

Sleep quality is also associated with chronotype: individual differences in time-of-day preference [37], [38]. It has been found that evening-types (who prefer later bed and rising times), have less regular sleeping habits, less time in bed during the week, and more time in bed at weekends [39], [40]. Poorer subjective sleep quality and more daytime sleepiness are also more frequent in evening-types [41], [42], [43], who also consume more alcohol, caffeine, and tobacco [39], [41], [43], [44] and report more emotional and psychological problems, such as anxiety and depression [38], [42]. Furthermore, eveningness has been found to correlate with more mind wandering and daydreaming [33].

In contrast to these findings, morning-preference is associated with more positive affect [45] and also better sleep quality and more mindfulness [30]. However, Howell et al. [30] found that the correlation between morning-preference and mindfulness was no longer significant after controlling for sleep quality. This suggests that sleep quality could be a mediator in the relationship between eveningness and mind wandering/daydreaming. However, mindfulness and mind wandering are not directly opposite states. Mindfulness does not necessarily imply an absence of thoughts, instead involving awareness of present thoughts as passing events, without becoming attached to them [27]. Mindfulness also involves meta-awareness, which may or may not be present during mind wandering [46], [47]. It remains to be tested whether sleep quality is involved in the relationship between chronotype and mind wandering in a similar way that it may be in the relationship between chronotype and mindfulness. Negative affect, as a shared correlate of these variables, may also be involved.

In summary, there is some evidence linking mind wandering and daydreaming with sleep disturbance, but there is still a lack of research on how it is related to different aspects of sleep quality in everyday life. The present study aimed to investigated these relationships. Additionally, the influence of sleep quality on the associations between mind wandering/daydreaming and negative affect, and between mind wandering/daydreaming and chronotype, were explored.

## Method

### Participants

We report data from three surveys, which involved independent samples of participants who were given different sets of questionnaires. Participants were full-time or part-time students at two Beijing universities, taking undergraduate, post-graduate or short-term training courses.

For the first survey (*study 1*), questionnaires were distributed to 248 participants; after excluding missing data (see *Statistical Analysis*, below), the final sample consisted of 213 participants, 74 males, and 139 females, aged 18–41 (mean = 23.96, sd = 5.42; male mean = 27.03, female = 22.33; t = 6.095, p<.0005, two-tailed). A sub-group of this sample (N = 88) completed a retest approximately 5 weeks after the first survey. Complete sets of data were obtained from 75 participants, 14 male, 61 female, aged 18–25 (mean = 19.81, sd = 1.58; male mean = 19.50, female = 19.89; t = .819, p = .415, two-tailed).

For the second survey (*study 2*), questionnaires were distributed to 225 participants; after excluding missing data, the final sample consisted of 190 participants, 52 males, 138 females, aged 18–50 (mean = 23.76, sd = 6.75; male mean = 25.96, female = 22.93; t = 2.366, p = .021, two-tailed). A sub-group of this sample (N = 99) completed a retest approximately 5 weeks after the first survey. Complete sets of data were obtained from 89 participants, 19 males, 70 females, aged 18–24 (mean = 19.81, sd = 1.06; male mean = 20.00, female = 19.76; t = .881, p = .381, two-tailed).

For the the third survey (*study 3*) 296 undergraduates were given two sets of questionnaires seven days apart; the order of scales was varied for different groups of students to provide some degree of counter-balancing. Excluding missing data, the final sample consisted of 270 participants, 74 males and 196 females, aged 18–21 (mean = 18.90, sd = .79; male mean = 19.14, female = 18.81; t = 3.047, p = .003, two-tailed). A third set of questionnaires was administered to the same participants approximately 5–6 weeks after the second set. This included some scales for test-retest and some other scales not reported here. Complete sets of data for retest were obtained from 171 participants (42 male, 129 female), aged 18–21 (mean = 18.70, sd = .76; male mean = 19.0, female = 18.6; t = 2.995, p = .004, two-tailed).

### Ethics Statement

Ethical approval was obtained from the Psychology Institute of the Chinese Academy of Sciences. Participants gave written consent on the survey questionnaires. Participation was voluntary and unpaid.

### Materials

1. The Chinese language version of the reduced Morningness-Eveningness Questionnaire (rMEQ [48], [49]), derived from Li et al.’s [50] Chinese translation of Horne and Östberg’s [37] Morningness-Eveningness Questionnaire (MEQ). Scores on the rMEQ range 4–25, and can be grouped into three chronotype categories: evening (4–11), neutral (12–17), and morning (18–25). The Chinese rMEQ has shown consistency with the full MEQ, with evidence for convergent validity and acceptable test-retest reliability [33], [49].
2. The Chinese versions of the Daydream Frequency (DF), and Mind Wandering (MW) scales [33], taken from the Imaginal Process Inventory (IPI [51]). Both scales have 12 items, each with five response options, and a possible range of 12–60. Higher scores indicate more mind wandering or daydreaming. The Chinese versions have shown good internal consistency, test-retest reliability, and convergent validity [33]. Although these concepts are strongly related, ‘mind wandering’ may more effectively characterise the experience of having frequent shifts of attention, while ‘daydreaming’ may involve more sustained internal focus [3], [47].
3. The Chinese version of the Pittsburgh Sleep Quality Index (PSQI [52]) made by Liu et al. [53]. The PSQI assesses seven, equally weighted components of sleep quality, along with reported bed and rise times, and sleep duration, over the preceding month. Each component is scored on a scale of 0–3, where a higher score indicates more problems on the corresponding component (e.g., a score of 3 on the sleep duration component indicates less sleep than does a score of 2). Each component score is derived from one or more of the scale items. Summing the seven components gives a Global sleep quality score (range 0–21).
4. The Chinese version of the Epworth Sleepiness Scale (ESS [54]) made by Peng et al. [55]. The ESS requires participants to rate how likely they feel it is that they would doze/fall asleep in eight different everyday situations (e.g., while watching TV). A scale of 0–3 is used for each item, with a higher score indicating greater sleep propensity. Total scores range 0–24. For clarity, minor changes of wording were made to Peng et al.’s [55] version.
5. The Chinese version of the Positive and Negative Affect Schedule (PANAS [56]) made by Huang et al. [57]. The scale has 10 items assessing positive affect (PA), and 10 items for negative affect (NA), both ranging 10–50. As Watson et al. ( [56], p.1063) state “High PA is a state of high energy, full concentration, and pleasurable engagement, whereas low PA is characterised by sadness and lethargy … Negative Affect … is a general dimension of subjective distress and unpleasurable engagement …” PA and NA can be assessed over different time-spans, ranging from ‘in general’ to ‘the present moment’. The current research stated ‘the last 3–4 weeks’, to make it consistent with the time-span of the PSQI.
6. A new Chinese translation of the Problem-Solving Daydreams (PS-DD) scale from the Imaginal Process Inventory (IPI [51]), which was designed to establish “…what role daydreams might play in practical problem solution…” ( [51], p.190), with items such as “My daydreams are closely related to problems that come up during my daily life” (item 5), and “Sometimes an answer to a difficult problem will come to me during a daydream” (item 10). The translation was made using the back-translation method; a native Chinese speaker made the initial translation, which was back-translated by another native Chinese speaker, and then checked by a native English speaker. Discrepancies were discussed and appropriately modified. This scale has 12 items, each with five response options (range 12–60).

### Procedure

The three surveys comprised different combinations of the above scales, plus demographics (and some other scales not reported here). Studies 1 and 2 were done contemporaneously and investigated associations between daydreaming, mind wandering and aspects of sleep quality. Study 1 involved the PSQI, rMEQ, and DF scales; study 2 involved the PSQI, rMEQ, and MW scales. Study 3 aimed to replicate and extend the findings of studies 1 and 2. In addition to the PSQI, MW, DF and rMEQ scales, it included the ESS, PANAS and PS-DD scales. The latter was included based on unpublished survey results which suggested a possible connection with sleep quality. Of the 171 participants involved in the study 3 retest, all completed the DF, PANAS and PSQI scales, and 74 additionally completed the MW scale (one extreme outlier was removed, leaving n = 73).

The sequence of the scales was varied to some extent in each survey, to provide some degree of counter-balancing. The surveys were completed by participants during class breaks, at various times of day, between April-May 2013 (studies 1 and 2) and October-November 2013 (study 3).

### Statistical Analysis

Regarding missing data, a single missing item from the DF, MW, PANAS-NA, PANAS-PA, ESS, or PS-DD scales was replaced by the mean of the other responses for that participant. Questionnaires were excluded if there were two or more omissions, or an error. For the rMEQ scale, those with an error or one/more omissions, were excluded. For the PSQI, because of the different kinds of calculations required for each component, a set of rules was established to deal with missing or ambiguous data; these are available from the authors. Cases with missing scores for one/more of the seven PSQI components were omitted.

Given the exploratory aims of this research much of the analysis was descriptive in nature, focusing on findings that were replicated across two or all three of the studies. We report zero-order Pearson product-moment correlations (two-tailed), partial correlations controlling for the influence of age, and regression analyses to test for significant predictors of the main variables. Also, following the procedures suggested by Preacher and Hayes [58], [59], mediation analyses tested whether sleep quality mediates a) the relationships between mind wandering and negative affect, and daydreaming and negative affect; and, b) the relationships between chronotype and mind wandering, and chronotype and daydreaming.

## Results

The psychometric properties of the scales for all studies are shown in Table 1. The scales showed reasonable/good reliability (assessed by Cronbach’s alpha, and/or test-retest). The values of Cronbach’s alpha for the 7 components of the PSQI were moderate, but similar to those reported in other studies; for example, .56 [63] and .66 [62]. Although the Shapiro-Wilk test indicated deviation from normality in most cases, the scores for each scale approximated a normal distribution.

**Table 1:** Psychometric properties of each scale, for each study.

	Scale range (possible)	Scale mean (sd)	Cronbach’s alpha	Mean inter-item correlation	Skew/Kurtosis		Shapiro-Wilk (statistic/df/p-value)	Test-retest co-efficient†
*Study 1 (N = 213)*								
DF	12–59 (12–60)	33.51 (9.54)	.906	.459	.295/−.289		.987/213/.048	.828
rMEQ	8–21 (4–25)	14.76 (3.03)	.619	.233	−.072/−.738		.976/213/.001	.670
PSQI Global	0–16 (0–21)	5.84 (2.57)	.664*	.210	.646/.958		.964/213/p<.0005	.770
*Study 2 (N = 190)*								
MW	16–52 (12–60)	35.66 (6.20)	.822	.284	−.264/.085		.991/190/.321	.722
rMEQ	6–21 (4–25)	14.48 (3.15)	.604	.215	−.076/−.205		.982/190/.017	.863
PSQI Global	0–14 (0–21)	5.83 (2.31)	.581*	.160	.435/.984		.961/190/p<.0005	.539
*Study 3 (N = 270)*								
MW	15–60 (12–60)	36.16 (6.81)	.881	.387	.096/.992		.986/270/.012	.691
DF	17–60 (12–60)	34.44 (8.82)	.865	.365	.294/−.373		.985/270/.006	.706
PS-DD	18–60 (12–60)	37.64 (6.56)	.849	.329	.339/1.153		.982/270/.001	−
rMEQ	6–23 (4–25)	14.34 (2.71)	.558	.191	.219/.194		.982/270/.002	−
ESS	2–21 (0–24)	11.23 (3.52)	.664	.205	−.074/.078		.990/270/.053	−
PANAS PA	15–50 (10–50)	31.37 (5.87)	.842	.374	.116/.373		.990/270/.06	.603
PANAS NA	10–46 (10–50)	21.67 (6.56)	.856	.373	.775/.609		.957/270/p<.0005	.602
PSQI Global	1–13 (0–21)	5.82 (2.19)	.576*	.161	.464/.317		.967/270/p<.0005	.613

DF = Daydream Frequency; MW = Mind Wandering; rMEQ = reduced Morningness-Eveningness Questionnaire;

PSQI Global = Global PSQI score (the sum of the seven components); PS-DD = Problem-Solving Daydreams;

ESS = Epworth Sleepiness Scale; PANAS = Positive and Negative Affect Schedule; PA = Positive Affect;

NA = Negative Affect.

† Pearson, two-tailed; study 1, N = 75; study 2, N = 89; study 3, N = 171 (except MW = 73); all *ps*<.0005.

*Based on the seven separate components.

Chronotype classifications for each study, with scale means for each chronotype, are shown in Table 2. Across the 3 studies, evening-types consistently had higher means for mind wandering or daydreaming (cf. [33]), and poorer sleep quality, shown by the higher PSQI Global score (cf. [41]). Separate multiple regression analyses with age and gender as predictor variables were done for the PSQI, rMEQ, DF, MW, ESS, PANAS, and PS-DD scales. Significant predictors are shown in Table 2. There was a significant gender difference for DF in study 1 (higher mean DF score for females; cf. [27]), but not in study 3; further study of gender differences seems required. Negative associations between age and DF, and age and MW were found in studies 1 and 2, which both had a wider age range than did study 3.

**Table 2:** Chronotype classifications, and descriptive statistics by chronotype.

	Chronotype classification			Age β	Gender β
	evening-type	neutral-type	morning-type		
*Study 1 (N = 213)*					
*Chronotype*	33 (15.5%)(14/42.4% male)	133 (62.4%)(44/33.1% male)	47 (22.1%)(16/34% male)	.215**	.194** (male mean = 14.32; female = 14.99)
Age †	24.06 (6.15)‡	23.31 (5.01)	25.74 (5.69)	−	−
Daydrea m Frequency	34.82 (8.38)‡	34.56 (9.44)	29.62 (9.74)	−.225**	.148* (male mean = 30.36; female = 35.19)
PSQI Global	6.70 (3.08)‡	5.86 (2.45)	5.17 (2.37)	(Component 6,.217**)(Component 7, −.203**)	
*Study 2 (N = 190 )*					
Chronotype	28 (14.7%)(9/32.1% male)	126 (66.3%)(35/27.8% male)	36 (18.9%)(8/22.3% male)	.211**	
Age †	22.36 (5.88)‡	23.37 (6.27)	26.25 (8.41)	−	−
Mind Wandering	37.04 (7.89)‡	35.94 (5.86)	33.64 (5.59)	−.143 (p = .054)	
PSQI Global	6.32 (2.31)‡	5.87 (2.28)	5.28 (2.37)		
*Study 3 (N = 270)*					
Chronotype	36 (13.3%)(13/36.1% male)	201 (74.4%)(55/27.4% male)	33 (12.2%)(6/18.2% male)		.140* (male mean = 13.69; female = 14.59)
Age †	19.08 (.84)‡	18.89 (.74)	18.79 (1.02)	−	−
Daydrea m Frequency	37.11 (8.61)‡	34.2 (7.99)	33.00 (7.89)		
Mind Wandering	39.14 (8.29)‡	36.05 (6.14)	33.61 (7.90)		
PSQI Global	7.08 (2.32)‡	5.62 (2.07)	5.70 (2.38)	.116 (p = .063)(Component 2,.203**)	
Epworth Sleepiness Scale	10.53 (3.40)‡	11.46 (3.37)	10.58 (4.42)		.113 (p = .063) (male mean = 10.58; female = 11.47)
Problem-solving Daydreams	38.06 (6.95)‡	37.37 (6.48)	38.82 (6.66)		
Positive Affect	29.81 (5.53)‡	31.20 (5.85)	34.15 (5.60)		
Negative Affect	22.61 (5.90)‡	21.59 (6.80)	21.09 (5.79)	.181**	

† See Methods section for gender differences.

‡ Mean (standard deviation). *p≤.05; **p≤.01.

Means and standard deviations for bed and rise times, sleep duration (from reported times, before coding into component 3), and for each PSQI component are shown in Table 3. A high percentage of participants in each study reported a PSQI Global score >5, indicating poor quality sleep [52]. Similar component mean values, and mean rankings, were reported across the three studies. For studies 1 and 2, ranking highest to lowest were components 7, 1, 5, 2, 3, 4, 6; for study 3 the ranks were: 7, 1, 3, 2, 5, 4, 6. These ranks (particularly the higher rank for component 7, and lower ranks for components 4 and 6) are similar to those found in some other studies (e.g., [60], [52]). In all three studies, very few participants reported using sleep medication (component 6): only 5/213 (2.3%) in study 1; 5/190 (2.6%) in study 2; and 2/270 (.7%) for study 3, meaning that nearly all participants had a score of zero for this component. Scores for component 4 (sleep efficiency) were also quite restricted in range, with >80% of participants in each study scoring zero (most efficient). The PSQI scoring system [52] gives a sleep efficiency score of zero for reported sleep time that is >85% of reported time-in-bed. Frequently reported responses, such as 8 hours in bed with 7 hours sleep ( = 87.5%), or 7 hours in bed with 6 hours sleep ( = 85.7%), met this criterion.

**Table 3:** Descriptive statistics for each PSQI component for each study.

	Mean score			Test-retest coefficient†		
	Study 1(N = 213)	Study 2(N = 190)	Study 3(N = 270)	Study 1(N = 75)	Study 2(N = 89)	Study 3(N = 171)
Bed time ‡ (sd/range)	23∶50(56/21∶30–02∶30)	23∶53(48/22∶00–02∶00)	24∶05(40/22∶30–03∶00)	.735***	.713***	.633***
Rise time ‡ (sd/range)	07∶12(40/05∶00–10∶30)	07∶20(47/05∶00–10∶00)	07∶14(40/06∶00–10∶00)	.836***	.766***	.648***
Sleep duration ‡ (sd/range in hours)	06∶47 (55/4.5–10)	06∶47 (50/4.5–10)	06∶40 (46/3.5–9)	.584***	.580***	.605***
Component 1 Subjective sleep quality (sd)	1.04 (.67)	1.00 (.65)	1.02 (.68)	.590***	.431***	.502***
Component 2 Sleep latency (sd)	.96 (.90)	.88 (.83)	.87 (.88)	.762***	.538***	.636***
Component 3 Sleep duration (sd)	.85 (.60)	.88 (.56)	.92 (.48)	.579***	.421***	.414***
Component 4 Habitual sleep efficiency (sd)	.17 (.45)	.21 (.46)	.15 (.43)	.368**	.304**	.052
Component 5 Sleep disturbances (sd)	.98 (.48)	.99 (.48)	.84 (.43)	.189	.428***	.310***
Component 6 Medication use (sd)	.03 (.19)	.03 (.20)	.01 (.09)	−	−	−.015
Component 7 Daytime dysfunction (sd)	1.81 (.87)	1.84 (.86)	2.02 (.76)	.505***	.591***	.488***
Global Score (sd)	5.84 (2.57)	5.83 (2.31)	5.82 (2.19)	.770***	.539***	.613***
% PSQI Global score >5 (male/female)	49.3 (51.4/48.2)	56.8 (57.7/56.5)	52.2 (52.7/52)	−	−	−

† Pearson, two-tailed.

*p≤.05;

**p≤.01;

***p≤.0005.

‡ Clock times; sd in minutes.

Correlations between bed, rise and sleep times, PSQI components, daydream frequency (DF), mind wandering (MW) and chronotype (rMEQ), are shown in Table 4. In both studies 1 and 3, DF significantly correlated with PSQI Global score and PSQI component 1 (subjective sleep quality), component 2 (sleep latency), component 5 (sleep disturbances) and component 7 (daytime dysfunction). The same pattern of significant correlations was shown for MW across studies 2 and 3, in addition to significant correlations with bed time (later bed time associated with more mind wandering). In all cases the positive correlations indicate that more mind wandering and daydreaming are associated with more sleep problems (poorer sleep quality, longer sleep latency, more sleep disturbance and more daytime dysfunction).

**Table 4:** PSQI correlations with daydreaming, mind wandering and chronotype.

	*Daydream Frequency*		*Mind Wandering*		*Chronotype (rMEQ)*		
*PSQI*	Study 1	Study3	Study 2	Study 3	Study 1	Study 2	Study 3
Bed time	.165* †	.085	.146*	.190**	−.507***	−.485***	−.369***
Rise time	.103	.064	.025	.122*	−.242***	−.234**	−.245***
Sleep duration ‡	−.105	−.109	−.028	−.145*	.298***	.124	.146*
C1 Subjective sleep quality	.167*	.178**	.291***	.227***	−.184**	−.127	−.108
C2 Sleep latency	.202**	.255***	.167*	.272***	−.103	−.098	−.029
C3 Sleep duration ‡	.075	.084	.021	.136*	−.265***	−.044	−.123*
C4 Habitual sleep efficiency	.108	.142*	−.115	.131*	−.028	.069	−.087
C5 Sleep disturbances	.209**	.150*	.183*	.165**	−.07	−.047	−.023
C6 Use of medication	−.110	−.024	.001	−.006	.104	.027	.090
C7 Daytime dysfunction	353***	.321***	.375***	.364***	−.262***	−.187**	−.209**
Global PSQI score	.299***	.344***	.302***	.394***	−.244***	−.145*	−.163**
Chronotype (rMEQ)	−.165*	−.119 (p = .052)	−.190**	−.207**	−	−	−

Study 1, N = 213; Study 2, N = 190; Study 3, N = 270.

*p≤.05;

** p≤.01;

*** p≤.0005.

† Partial correlations, controlling for age.

‡ ‘Sleep duration’ = reported hours of sleep; ‘C3 Sleep duration’ = coded, whereby higher score = less sleep duration.

Across studies 1–3, rMEQ showed consistent correlations with bed and rise times, such that morning-types go to bed and rise earlier than do evening-types. Across the three studies, rMEQ showed significant negative correlations with daydream frequency and mind wandering (cf. [33]); rMEQ was also inconsistently correlated with subjective sleep quality, and with sleep duration, and consistently correlated with PSQI Global, and strongly with PSQI component 7 (daytime dysfunction). When component 7 was removed from the PSQI Global score, two of the correlations with rMEQ (all partialled for age) were no longer significant: study 1, r = −.188 (p = .006); study 2, r = −.093 (p = .204); study 3, r = −.105 (p = .084).

PSQI component 5 is comprised of nine aspects of sleep disturbance (questions 5b–5j), and component 7 is comprised of question 8 (“During the past month, how often have you had trouble staying awake while driving, eating meals, or engaging in social activity?”), and question 9 (“During the past month, how much of a problem has it been for you to keep up enough enthusiasm to get things done?”). Correlations between these items and DF, MW, and rMEQ, are shown in Table 5. Across the three studies, DF, MW, and rMEQ were each consistently correlated with PSQI questions 8 and 9; DF was consistently correlated with question 5c (having to get up to use the bathroom), and with question 5f (feel too cold); DF, like MW, was also inconsistently correlated with question 5g (feel too hot). Correlations with questions 5j were somewhat inconsistent, but this is an open-ended question. Frequent answers among younger, undergraduate participants, were stress related to study, and noisy (dormitory) environment (cf. [19]).

**Table 5:** Correlations with PSQI items 5b–5j, 8 and 9.

	*Daydream Frequency*		*Mind Wandering*		*Chronotype (rMEQ)*		
*PSQI item*	Study 1	Study 3	Study 2	Study 3	Study 1	Study 2	Study 3
5b Wake up in the middle of the night or early morning	.146* †	.103	.140	.116	.041	−.009	.019
5c Have to get up to use the bathroom	.236**	.153*	−.075	.067	.008	.096	.066
5d Cannot breathe comfortably	.101	.028	.050	.078	−.013	.023	−.083
5e Cough or snore loudly	.027	.064	.001	.105	−.108	−.064	−.125*
5f Feel too cold	.263***	.191**	.184*	.112	−.105	−.028	−.003
5g Feel too hot	.045	.159**	.111	.183**	−.059	−.089	−.042
5h Had bad dreams	.100	.125*	.194**	.030	−.025	−.134	−.029
5i Have pain	.177**	.013	.160*	−.009	−.157*	.048	.013
5j Other	.128	.084	.188**	.102	−.094	−.078	−.034
8 Trouble staying awake	.323***	.342***	.389***	.356***	−.286***	−.180*	−.213***
9 Lack enthusiasm to get things done	.319***	.281***	.303***	.396***	−.179**	−.166*	−.185**

Study 1, N = 213; Study 2, N = 190; Study 3, N = 270.

*p≤.05;

** p≤.01;

*** p≤.0005.

† Partial correlations, controlling for age.

PSQI item 8 suggests daytime sleepiness, while question 9 suggests lack of energy or motivation, which may involve an affective component (these questions correlated .561; p<.0005). To explore these influences, study 3 included specific measures of daytime sleepiness (the ESS) and affect (the PANAS). Correlations are shown in Table 6 (see also Table 4). Substantial daytime sleepiness, as defined by a score >10 [64] was reported by 57.8% of participants (48.6% of males; 61.2% of females). Both daydream frequency and mind wandering showed significant positive correlations with the ESS, consistent with their positive correlations with PSQI component 7. However, rMEQ did not correlate with the ESS total score (or with any of the items, all *rs* <.1). Also, Problem-Solving Daydreams were uncorrelated with DF and with PSQI Global score, but negatively correlated with MW, the ESS, and PSQI component 7 (r = −.134; p = .028).

**Table 6:** Correlations with daytime sleepiness, positive affect, negative affect, and problem-solving daydreams.

	Epworth Sleepiness Scale	Problem-Solving Daydreams	Positive Affect (PANAS)	Negative Affect (PANAS)
Chronotype (rMEQ)	−.009†	.054	.214***	−.038
Daydream Frequency	.246***	−.081	−.326***	.412***
Mind Wandering	.331***	−.247***	−.377***	.333***
PSQI Global score	.206**	−.044	−.332***	.367***
Epworth Sleepiness Scale	−	−.214***	−.134*	.174**
Problem-solving Daydreams	−	−	.230***	.034

† Partial correlations, controlling for age. N = 270.

*p≤.05;

**p≤.01;

***p≤.0005.

Morningness (rMEQ) correlated with positive affect (PA), but not with negative affect (NA), consistent with other findings [45], as are the correlations between poor sleep and more negative affect, and less positive affect [19], [61]. PA-NA correlated −.153 (p = .012; cf. Watson et al. [56], who reported a range of −.12 to −.23). DF, MW, PSQI Global, and ESS showed the same significant pattern of negative correlation with PA, and positive correlation with NA. MW and DF correlated .644 (p<.0005; cf. Carciofo et al. [33], r = .573).

### Test-retest Analyses

The test-retest coefficients for the Global PSQI scores were moderate/good, but there was quite a range in the component coefficients (Table 3). Coefficients could not be calculated for component 6 in studies 1 and 2 because all participants had a score of zero in test 1 (*T1*) and/or test 2 (*T2*), and >98% of participants (in *T1* and *T2)* had a score of zero in study 3. Buysse et al. [52] also reported no correlation for component 6 (in non-clinical participants; coefficient not reported). Scores for component 4 were also restricted (>80% in all studies scoring zero in both tests), and the test-retest coefficients were modest. Component 5 also showed variable test-test coefficients, but as Buysse et al. [52] note, this component includes many aspects of sleep which may vary over time.

Regression analyses tested whether *T1* variables (PSQI components, MW and DF) predicted retest/*T2* variables, while controlling for the dependent variable at *T1*. In study 1, *T1* DF did not predict any *T2* PSQI components, and *T1* PSQI components did not predict *T2* DF (all *ps*>.05). In study 2, *T1* PSQI components did not predict *T2* MW (all *ps*>.05), but *T1* MW predicted *T2* PSQI components 5 (β = .256, p = .008), 7 (β = .314, p = .001), and Global (β = .299, p = .002). However, in study 3, *T1* MW (or DF) did not significantly predict any *T2* PSQI components, and *T1* PSQI components did not predict *T2* MW (or DF). It seems that there are no consistent predictive relationships between DF/MW and PSQI components at the test-retest interval used in these studies (5–6 weeks).

### Mediation Analyses

#### 1) Mind wandering, negative affect, and sleep quality

**Figure 1:**
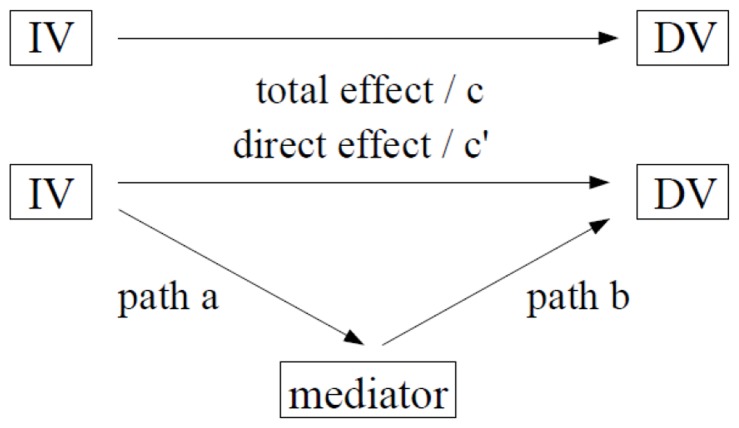
Paths of mediation analysis.

Much recent research has discussed the associations between mind wandering or daydreaming, and negative affect, suggesting a bi-directional relationship. Study 3 established mutual associations between poor sleep quality, negative affect, and both mind wandering and daydreaming, so a possible mediating role for sleep quality (while controlling for age and gender), was tested using the procedures suggested by Preacher and Hayes [58], [59]. This involves (see Figure 1):- 1) *path c* - the total effect of the IV/predictor on the DV/criterion, excluding the proposed mediator (although this path does not need to be significant for a test of mediation); 2) *path c’* - the direct effect of the IV on the DV (testing if this remains significant while controlling for the mediator); 3) *path a* - the effect of the IV on the proposed mediator; 4) *path b* - the direct effect of the mediator on the DV (testing if this is significant while controlling for the IV); and, 5) path a*b - the indirect effect of the mediator through the IV. The significance of this indirect path was tested with a non-parametric bootstrapping procedure in which 5000 re-samples were taken from the data to establish 95% confidence intervals, with significance indicated by the exclusion of zero [58], [59].

The results are shown in Table 7. With negative affect (NA) as the predictor for DF (model 1a) and for MW (model 1b), sleep quality (PSQI Global score) was found to have significant direct and indirect effects in both cases. However, NA also retained a significant direct effect in both models, indicating that sleep quality is a partial mediator of the relationships between negative affect and mind wandering, and negative affect and daydreaming. When the relationship was reversed, so that DF and MW were the predictors for NA (models 2a and 2b), the same pattern was found, with sleep quality having significant direct and indirect effects, but DF and MW also retaining significant direct effects.

**Table 7:** Sleep quality as a mediator in the relationships between negative affect and daydreaming, and negative affect and mind wandering.

*Model 1a*							
	IV	mediator	DV	IV β	Mediator β	Age β	Gender β
Path c	NA	−	DF	.418***	−	−.037	−.076
Path a	NA	PSQI Global	−	.371***	−	.048	.035
Paths b and c’	NA	PSQI Global	DF	.333***	.227***	−.048	−.084
Final Model: R = .471; adjusted R^2^ = .210; *F*(4, 265) = 18.849; Indirect effect (a * b): bias corrected† 95% C.I. = .0432 to .1849							
***Model 1b***							
	**IV**	**mediator**	**DV**	**IV β**	**Mediator β**	**Age β**	**Gender β**
Path c	NA	−	MW	.338***	−	.001	−.018
Path a	NA	PSQI Global	−	.371***	−	.048	.035
Paths b and c’	NA	PSQI Global	MW	.220***	.316***	−.014	−.029
Final Model: R = .448; adjusted R^2^ = .188; *F*(4, 265) = 16.600; Indirect effect (a * b): bias corrected 95% C.I. = .0667 to .1985							
***Model 2a***							
	**IV**	**mediator**	**DV**	**IV β**	**Mediator β**	**Age β**	**Gender β**
Path c	DF	−	NA	.407***	−	.165**	.024
Path a	DF	PSQI Global	−	.347***	−	.102	.06
Paths b and c’	DF	PSQI Global	NA	.320***	.252***	.140*	.009
Final model: R = .503; adjusted R^2^ = .242; *F*(4, 265) = 22.441; Indirect effect (a * b): bias corrected 95% C.I. = .0322 to .1267							
***Model 2b***							
	**IV**	**mediator**	**DV**	**IV β**	**Mediator β**	**Age β**	**Gender β**
Path c	MW	−	NA	.328***	−	.161**	−.002
Path a	MW	PSQI Global	−	.393***	−	.091	.04
Paths b and c’	MW	PSQI Global	NA	.219***	.277***	.135*	−.013
Final model: R = .452; adjusted R^2^ = .192; *F*(4, 265) = 17.018; Indirect effect (a * b): bias corrected 95% C.I. = .0525 to.1716							

NA = Negative Affect; DF = Daydream Frequency; PSQI Global = PSQI Global score; MW = Mind Wandering.

† Taking account of the difference between the initial and the mean indirect effect estimates.

*p≤.05; **p≤.01; ***p≤.0005.

#### 2) Mind wandering, daydreaming, sleep quality and chronotype

Mediation analysis was also undertaken to test whether sleep quality significantly mediates the relationship between chronotype (rMEQ) and daydream frequency, and between chronotype and mind wandering (while controlling for age and gender). Scores for PSQI component 7 were used as the mediator, because, as noted above, this was the only component to consistently correlate with rMEQ, and removing this from the PSQI Global score substantially reduced the correlations between rMEQ and PSQI Global (results using PSQI Global were similar, but the mediation effect was slightly weaker). Results are shown in Table 8.

**Table 8:** Sleep quality as a mediator between chronotype and daydreaming, and chronotype and mind wandering.

*Study 1; Dependent variable = Daydream Frequency*							
	IV	mediator	DV	IV β	Mediator β	Age β	Gender β
Path c	rMEQ	−	DF	β = −.190**	−	−.184*	.185*
Path a	rMEQ	C7	−	−.261***	−	−.147*	.002
Paths b and c’	rMEQ	C7	DF	−.106 (p = .103)	.323***	−.137*	.185**
Final model: R = .478; adjusted R^2^ = .214; *F*(4, 208) = 15.423; Indirect effect (a * b): bias corrected† 95% C.I. = −.4877 to −.1190							
***Study 3; Dependent variable = Daydream Frequency***							
	**IV**	**mediator**	**DV**	**IV β**	**Mediator β**	**Age β**	**Gender β**
Path c	rMEQ	−	DF	−.110 (p = .075)	−	.034	−.064
Path a	rMEQ	C7	−	−.212**	−	.041	.021
Paths b and c’	rMEQ	C7	DF	−.044 (p = .463)	.311***	.021	−.071
Final model: R = .336; adjusted R^2^ = .100; *F*(4, 265) = 8.445; Indirect effect (a * b): bias corrected 95% C.I. = −.3612 to −.0858							
***Study 2; Dependent variable = Mind Wandering***							
	**IV**	**mediator**	**DV**	**IV β**	**Mediator β**	**Age β**	**Gender β**
Path c	rMEQ	−	MW	−.201**	−	−.100	.111
Path a	rMEQ	C7	−	−.202**	−	−.049	.135
Paths b and c’	rMEQ	C7	MW	−.132 (p = .059)	.341***	−.083	.065
Final model: R = .426; adjusted R^2^ = .164; *F*(4, 185) = 10.250; Indirect effect (a * b): bias corrected 95% C.I. = −.2595 to −.0455							
***Study 3; Dependent variable = Mind Wandering***							
	**IV**	**mediator**	**DV**	**IV β**	**Mediator β**	**Age β**	**Gender β**
Path c	rMEQ	−	MW	−.208**	−	.052	.009
Path a	rMEQ	C7	−	−.212**	−	.041	.021
Paths b and c’	rMEQ	C7	MW	−.138*	.335***	.039	.002
Final model: R = .392; adjusted R^2^ = .141; *F*(4, 265) = 12.053; Indirect effect (a * b): bias corrected 95% C.I. = −.3285 to −.0781							

DF = Daydream frequency; MW = Mind Wandering; rMEQ = reduced Morningness-Eveningness Questionnaire;

C7 =  PSQI component 7.

† Taking account of the difference between the initial and the mean indirect effect estimates.

*p≤.05; **p≤.01; ***p≤.0005.

*Daydreaming:* For study 1, PSQI component 7 had significant direct and indirect effects, and the direct effect of rMEQ (controlling for component 7) was not significant. For study 3, component 7 again had significant direct and indirect effects, while the total effect of rMEQ (excluding component 7) was only marginally significant (p = .075), and the direct effect (controlling for component 7) was not significant (p = .463).

*Mind Wandering:* For study 2, PSQI component 7 had significant direct and indirect effects. The direct effect of rMEQ (controlling for component 7) was also marginally significant (p = .059). For study 3, component 7 again had significant direct and indirect effects, and the direct effect of rMEQ (controlling for component 7) was also significant (p = .019).

The above analyses show that, in all cases, PSQI component 7 (daytime dysfunction), had significant direct and indirect effects on DF and MW. The direct effect of rMEQ on DF was marginal or non-significant, while it remained (marginally) significant for MW.

*Positive Affect:* Study 3 found that positive affect negatively correlated with mind wandering, daydreaming and sleep quality, but positively correlated with morningness. To explore the possible influence of positive affect, a further test was done in which both positive affect and sleep quality (PSQI Global) were included as mediators between chronotype (IV) and mind wandering/daydreaming (DV). As shown in Table 9, both positive affect and sleep quality had significant direct and indirect effects on the relationships between chronotype and daydreaming, and chronotype and mind wandering. Furthermore, with the inclusion of both mediators, the direct effect of rMEQ on DF was no longer significant (p = .897). A very similar result was obtained when PSQI component 7 was used as the mediator rather than PSQI Global (rMEQ β = .008, p = .889). For MW, when PSQI Global was used as the mediator, the direct effect of rMEQ on MW remained somewhat marginally significant (p = .084), but this was weaker when component 7 was used as the mediator instead of PSQI Global (rMEQ β = −.081, p = .154).

**Table 9:** Mediation analyses for chronotype, daydream frequency, mind wandering, sleep quality and positive affect.

*Dependent variable = Daydream Frequency*											
	IV	Mediator 1	Mediator 2	DV	IV β	Mediator 1 β		Mediator 2 β	Age β		Gender β
Path c	rMEQ	−	−	DF	−.110	−		−	.034		−.064
Path a1	rMEQ	PSQI Global	−	−	−.170**	−		−	.108		.056
Path a2	rMEQ	−	PA	−	.232***	−		−	−.043		−.128*
Paths b1, b2 & c’	rMEQ	PSQI Global	PA	DF	−.008 (p = .897)	.266***		−.246***	−.006		−.110
Final model: R = .428; adjusted R^2^ = .167; *F*(5, 264) = 11.816 Indirect effects: PSQI Global: bias corrected† 95% C.I. = −.2853 to −.0355; PA: bias corrected 95% C.I. = −.3178 to −.0758											
***Dependent variable = Mind Wandering***											
	**IV**	**Mediator 1**	**Mediator 2**	**DV**	**IV β**	**Mediator 1 β**		**Mediator 2 β**	**Age β**		**Gender β**
Path c	rMEQ	−	−	MW	−.208**	−		−	.052		.009
Path a1	rMEQ	PSQI Global	−	−	−.170**	−		−	.108		.056
Path a2	rMEQ	−	PA	−	.232***	−		−	−.043		−.128*
Paths b1, b2 & c’	rMEQ	PSQI Global	PA	MW	−.098 (p = .084)	.293***		−.263***	.009		−.041
Final model: R = .488; adjusted R^2^ = .224; *F*(5, 264) = 16.542 Indirect effects: PSQI Global: bias corrected 95% C.I. = −.2597 to −.0409; PA: bias corrected 95% C.I. = −.2828 to −.0683											
***Dependent variable = Chronotype***											
	**IV**	**Mediator 1**	**Mediator 2**	**DV**	**IV β**	**Mediator 1 β**		**Mediator 2 β**	**Age β**		**Gender β**
Path c	DF	−	−	rMEQ	−.108	−		−	−.042		.132*
Path a1	DF	PSQI Global	−	−	.347***	−		−	.102		.06
Path a2	DF	−	PA	−	−.335***	−		−	−.041		−.122*
Paths b1, b2 & c’	DF	PSQI Global	PA	rMEQ	−.008 (p = .897)	−.101 (p = .121)		.193**	−.024		.161**
Final model: R = .293; adjusted R^2^ = .068; *F*(5, 264) = 4.949 Indirect effects: PSQI Global: bias corrected 95% C.I. = −.0294 to .0042; PA: bias corrected 95% C.I. = −.0397 to −.0079											
***Dependent variable = Chronotype***											
	**IV**	**Mediator 1**	**Mediator 2**	**DV**	**IV β**	**Mediator 1 β**		**Mediator 2 β**	**Age β**		**Gender β**
Path c	MW	−	−	rMEQ	−.204**	−		−	−.034		.136*
Path a1	MW	PSQI Global	−	−	.393***	−		−	.091		.04
Path a2	MW	−	PA	−	−.380***	−		−	−.031		−.103
Paths b1, b2 & c’	MW	PSQI Global	PA	rMEQ	−.116 (p = .084)	−.068 (p = .298)		.162*	−.023		.156**
Final model: R = .310; adjusted R^2^ = .079; *F*(5, 264) = 5.606 Indirect effects: PSQI Global: bias corrected 95% C.I. = −.0337 to .0121; PA: bias corrected 95% C.I. = −.0477 to −.0061											

† Taking account of the difference between the initial and the mean indirect effect estimates.

*p≤.05;

**p≤.01;

***p≤.0005.

These findings support the model of evening-preference being associated with poorer quality sleep and less positive affect, which may contribute to the increased frequency of mind wandering/daydreaming. The reverse model is also possible, i.e., more mind wandering or daydreaming (IV) being associated with poorer quality sleep and less positive affect, which may predict more eveningness (DV). However, sleep quality was not a significant mediator in this model, for either DF or MW (Table 9).

Furthermore, exploratory analysis of these inter-relationships revealed that chronotype moderated the relationships between positive affect and daydreaming, and positive affect and mind wandering. This effect was shown in hierarchical regression, in which the product of Z-transformed positive affect (PA) and chronotype (rMEQ) scores, which were entered in the second step, produced significant changes in the models (Table 10).

**Table 10:** Moderation analysis: daydream frequency, mind wandering, chronotype and positive affect.

*Dependent Variable = Daydream Frequency*									
	rMEQ β	Positive Affect β	Age β	Gender β	rMEQ* PA β	R	R^2^ (adjusted)	*F* (df/residual)	change in R^2^
Step 1	−.034	−.328***	.019	−.106	−	.349	.121 (.108)	9.159*** (4, 265)	
Step 2	−.054	−.340***	.024	−.114	.173**	.388	.151 (.135)	9.365*** (5, 264)	.029**
***Dependent Variable = Mind Wandering***									
	**rMEQ β**	**Positive Affect β**	**Age β**	**Gender β**	PA*rMEQ β	**R**	**R^2^ (adjusted)**	***F*** ** (df/residual)**	**change in R^2^**
Step 1	−.126*	−.353***	.037	−.036	−	.405	.164 (.151)	12.981*** (4, 265)	
Step 2	−.142*	−.363***	.041	−.042	.133*	.426	.181 (.166)	11.675*** (5, 264)	.017*

*p≤.05; **p≤.01; ***p≤.0005.

For evening-types (n = 36) and neutral-types (n = 201), significant negative correlations were found between positive affect and daydreaming (evening-types: r = −.345, p = .039; neutral-types: r = −.364, p<.0005), and between positive affect and mind wandering (evening-types: r = −.379, p = .023; neutral-types, r = −.426, p<.0005). However, these correlations were not significant in morning-types (n = 33; DF: r = .05, p = .784; MW: r = −.004, p = .981).

## Discussion

### Sleep Quality and Chronotype

The current study, like some other recent studies (e.g., [19], [60], [62]), found that poor sleep quality is common in university students. High percentages of participants were classified as poor sleepers, using the criterion of a PSQI Global score >5 [52]: 49.3%, 56.8%, and 52.2%, for studies 1–3 respectively. Similarly, Suen et al. [63] found that 57.5% of a sample of 400 Hong Kong university students were classified as poor sleepers, while Lund et al. [19] found that >60% of a sample of 17–24 year old American students had PSQI Global scores >5 (see also [20], [60], [62]). Furthermore, 57.8% of our participants (in study 3) reported substantial daytime sleepiness, as defined by an Epworth Sleepiness Scale (ESS) score >10 [64], which is very high compared to some other studies (e.g., in a student sample, Lund et al. [19] found 25.5% scored 10/more). Consequently, the validity of the ESS responses may be questioned. However, the observed correlation between the ESS and PSQI Global score (r = .206) is similar to that reported by Buysse et al. ( [65] r = .160), who found that the scales are largely orthogonal. They also found PSQI component 7 (daytime dysfunction) to be the strongest association between the scales (r = .340), as did we (r = .291). Also, there were consistent inter-correlations between the ESS, DF, MW, PSQI and PANAS scales. Nevertheless, the current finding of a high percentage of reported daytime sleepiness stands in need of replication.

The chronotype classifications in studies 1 and 2 showed similar percentages to those found by Carciofo et al. [33], while the lower percentage of morning-types in study 3 likely reflects the narrower, younger age range in that study (18–21). Also, studies 1 and 3 found more morningness for females/more eveningness for males, consistent with other research [38]. Self-reported bed and rise times were consistent with chronotype classification, morningness being associated with earlier times. The observed correlation coefficients were similar to those reported by Barclay et al. [66] with the full MEQ, giving some convergent validity to the Chinese rMEQ. The correlation between eveningness and overall poorer sleep quality (higher PSQI Global score), is consistent with many other studies (e.g., [41], [42], [43], [66]). Both Vardar et al. [42] and Selvi et al. [67] found that evening-types reported poorer subjective sleep quality (component 1), more daytime dysfunction (component 7), and higher PSQI Global scores, as also found in the current study (although component 1 correlations were inconsistent). Although the Epworth Sleepiness Scale (ESS) did not correlate with chronotype, other studies have shown inconsistent findings for this association. For example, using the ESS, Vardar et al. [42], found that evening-types reported more daytime sleepiness, while Taillard et al. [39] found no significant differences between chronotypes.

### Mind Wandering, Daydreaming, Sleep Quality and Affect

Overall, reliable, moderate associations were found between poor sleep quality and the reported frequency of both mind wandering and daydreaming. Across the three studies, mind wandering and daydreaming both showed consistent, significant correlations with PSQI Global score, and in particular with PSQI component 1 (subjective sleep quality), component 2 (sleep latency), component 5 (sleep disturbances), and (especially strongly) with component 7 (daytime dysfunction). The negative correlation between PSQI component 7 and age found in study 1 was also reported by Buysse et al. [52], and age was also negatively correlated with daydreaming and mind wandering (in studies 1 and 2), as reliably found in other research (e.g., [33], [68], [69]). The negative correlation between self-reported hours of sleep per night and frequency of daydreaming reported by Kunzendorf et al. [13] was not consistently found in the current research. Correlations with sleep efficiency (component 4) were also inconsistent, while the correlations with component 6 were limited by the very low frequency of reported use of sleep medication. Finally, both mind wandering (MW) and daydreaming (DF) were positively correlated with daytime sleepiness as assessed with the Epworth Sleepiness Scale.

Although conclusions about causation cannot be drawn from this correlational data, these associations seem consistent with a bi-directional model, in which daydreaming and mind wandering may potentially be both a cause and a consequence of sleep problems/poor sleep quality. For example, the observed correlations between sleep latency and general mind wandering and daydreaming, are consistent with evidence showing that prolonged sleep latency or insomnia are related to worry and rumination [17], [18], [70], and also more general, non-ruminative mind wandering [22]. Furthermore, stress related to study was cited by some participants as a reason for sleep disturbance (component 5), as has been reported in other studies (e.g., [19]), which is consistent with much mind wandering or daydreaming being focused on an individual’s current concerns [1], [71]. The content of insomnia-related thoughts prior to sleep has been found to include such personal concerns [70]. Increased mind wandering late at night may also be consistent with the hyperarousal model of insomnia, which relates insomnia to daytime and night-time increases in biological and psychological arousal (see [35] for review). Buysse et al. [65] argue that this hyperarousal may be a common factor linking symptoms of poor sleep quality, stress, anxiety and depression, and it could also be that (some forms of) mind wandering or daydreaming are involved in this. Consistent with this possibility, there is some evidence linking mind wandering with increased arousal, as assessed by heart-rate (e.g., [22], [24]).

However, while mind wandering or daydreaming may be a cause of insomnia, they may also be a consequence of sleep disruption, as indicated in some previous studies (e.g., [16]). Some correlations with PSQI items related to sleep disturbance seem consistent with this. For example, daydreaming and/or mind wandering were (inconsistently) correlated with having sleep disturbed by feeling too hot or too cold, needing the bathroom, or having bad dreams. Such disturbances may also contribute to perceived poor sleep quality (PSQI component 1), which was consistently correlated with mind wandering and daydreaming.

However, for either direction of possible causation, any relationships between aspects of sleep quality, and mind wandering or daydreaming, need to be considered over specific time-frames. The current research did not find any consistent predictors (PSQI components predicting DF/MW, or vice versa), over the retest interval of 5–6 weeks. This may indicate that other variables are involved, or that the 5–6 week interval, and/or the retrospective assessment of general sleep quality and DF/MW, may be relatively insensitive measures. The effectiveness of shorter-term state measures is suggested by the finding that mind wandering assessed by thought-sampling during the day can predict sleep latency that evening [22]. Also, less sleep on the previous night is associated with reduced functional connectivity between neural circuits of the default mode network (DMN) and its anti-correlated network [72]. As the DMN is reliably associated with mind wandering [46], such sleep-related changes in its functioning could be further explored for their relationships to self-reported mind wandering or daydreaming frequency.

The basis of the relationship between mind wandering/daydreaming and daytime sleepiness could also be explored. Although daytime sleepiness is connected with lack of sleep [7], research evidence has not always supported a strong direct link. For instance, Alapin et al. [73] found that daytime sleepiness was not related to total sleep time or to sleep efficiency, and did not seem to be simply the result of insufficient sleep (the ESS correlations with sleep duration and sleep efficiency were also non-significant in the current research). It seems that an affective component may also be involved. Anxiety has been found to be a particularly strong predictor of insomnia, which in turn has been found to be a strong predictor of depression [34], [35]. Furthermore, in a large sample (>16,000), Bixler et al. [36] found that depression was the strongest predictor of excessive daytime sleepiness (EDS), while aspects of sleep disturbance were not significant. These findings suggest dynamic relationships between aspects of sleep quality and negative affect over an extended time period; forms of mind wandering or daydreaming may also be involved in these relationships.

Many previous studies have shown an apparently bi-directional relationship between mind wandering or daydreaming, and negative affect (e.g., [23], [25], [26], [27]). There is evidence that mindfulness is a mediator in the relationship between daydream frequency and psychological distress [27], and other variables may also be involved. For example, Mason et al. [74] suggest that possible ‘third variables’ may include stressful life events, and dispositional depression or neuroticism; inflexible, perseverative forms of thinking associated with rumination and worry may also be particularly related to negative affect [15]. The present research has contributed to this discussion by showing that the relationship between mind wandering/daydreaming and negative affect is partially mediated by sleep quality. Similarly, Howell et al. [30] found that although mindfulness directly predicted well-being, sleep quality was also a significant mediator. The relationships between mind wandering/daydreaming and negative affect/well-being seem to involve complex contextual and temporal influences [75]; the current research suggests that these influences include sleep quality.

### Implications for Theories of Mind Wandering

Acute or chronic sleep deprivation are known to have adverse effects on cognitive functioning, including impairment of executive functions [8], [9]. So, the finding that the frequencies of mind wandering and daydreaming are associated with poor sleep quality may support the view that mind wandering results from failures of executive control which allow spontaneous thoughts to enter consciousness [76]. In contrast, it could be argued that an emphasis on mind wandering as requiring executive resources [2] might imply that the frequency of mind wandering would be negatively correlated with poor sleep quality, as better sleep quality would improve the efficiency of cognitive functioning which could allow for more spare resources to be allocated to mind wandering. Instead, the observed positive correlation between poor sleep quality and mind wandering seems in some ways comparable to the effects of alcohol, which can produce similar cognitive impairments to those induced by sleep deprivation [7], [10], and which also increases mind wandering [77]. These considerations suggest that an increased frequency of intrusive episodes of mind wandering may occur due to impaired executive control [76], which may be a consequence of sleep disruption/poor quality sleep.

Mind wandering or daydreaming related to poor executive control may also be involved in causing sleep problems. Insomnia-related mind wandering [22] may be due to executive failure, allowing intrusive thoughts into consciousness. However, executive resources would also seem required for maintaining these thoughts within the ‘cognitive space’ [17], or ‘global conscious workspace’ [78], [79]. So, executive control failure and recruitment of executive resources may both be involved in sleep-related mind wandering or daydreaming. Further study of the reciprocal relationships between sleep quality and the initiation and maintenance of mind wandering or daydreaming episodes (distinguishing process and occurrence; [80]), may be insightful. The influence of poor sleep quality on the meta-awareness of mind wandering or daydreaming is also of interest; perhaps the overall frequency of mind wandering is increased, but the meta-awareness of particular episodes may be proportionately decreased, similar to the effects of alcohol on mind wandering [77].

Further consideration of more intentional and purposeful mentation, possibly related to the more adaptive functions of mind wandering or daydreaming [6], [78], [81], [82], may also help in elaborating the roles of executive control and executive resources. The present research found that the MW and DF scales were very similar in their correlations with NA, PA, PSQI Global, and the ESS, but the Problem-Solving Daydreams scale was negatively correlated with both MW and ESS, positively correlated with PA, and not correlated with DF, PSQI Global, rMEQ, or NA. Although the current findings should be replicated, they are consistent with some other research: Singer and Antrobus [51] reported that the Problem-Solving Daydreams scale was more associated with positive aspects of daydreaming, and Giambra and Traynor [23] found that the Problem-Solving Daydreams scale was uncorrelated with two questionnaire measures of depression, and negatively correlated with a third. The finding that the Problem-Solving Daydreams scale was uncorrelated with the Daydream Frequency scale, and negatively correlated with the Mind Wandering scale, suggests that this specific type of daydreaming is separate from the overall frequency of this class of mentation (at least as defined in the respective scales), and may be a useful measure of more goal-directed or purposeful daydreaming. A more fine-grained approach to different forms of mind wandering or daydreaming also helps to elucidate the relationships with affect, with evidence showing that some forms, or content (such as daydreaming of close family and friends [81]), are more associated with positive affect [6], [74], [82]. It may be that poor sleep quality is associated with more negatively valenced forms of mind wandering or daydreaming. Different ‘styles of daydreaming’ that vary in, for example, their controllability and acceptability to the individual [51], [83], [84], may also vary in their relationships with sleep quality.

### Mind Wandering, Daydreaming and Chronotype

One similarity between mind wandering and daydreaming (as measured by the MW and DF scales), is that the overall frequency of both was associated with more evening preference, replicating the finding of Carciofo et al. [33]. Also, across the three studies, mind wandering, daydreaming and chronotype (rMEQ) were consistently correlated with PSQI Global score, and particularly with component 7, daytime dysfunction (more eveningness, more DF and MW, associated with poorer quality sleep, especially in regard to daytime dysfunction). Howell et al. [30] reported that sleep quality seemed to mediate the correlation between morningness and mindfulness, and the present study showed that sleep quality (PSQI Global or component 7), at least partially mediated the relationships between rMEQ and DF, and rMEQ and MW. When positive affect (which was negatively correlated with eveningness, poor sleep quality, DF and MW) was included as an additional, second mediator, the direct effect of rMEQ on DF approached zero. The direct effect of rMEQ on MW also became non-significant, although somewhat marginal, suggesting that MW may be more strongly related to chronotype than is daydreaming (at least as they are measured with the corresponding scales).

The mediation analysis supported a model with chronotype as the predictor for daydreaming or mind wandering, suggesting that the association between evening chronotype and poor sleep quality may be related to evening-types having to rise at an earlier time than desired, due to work, school, or other social obligations, which may lead to the experience of more ‘social jet-lag’ [44]. Additionally, rising earlier than desired means that evening-types rise at a time closer to the nadir of the body temperature cycle (which is later in evening-types than morning-types), and so they may feel more sleepy and less alert, which could contribute to the perception of poorer sleep quality [66], and may contribute to less positive affect (less energy, enthusiasm, motivation, etc). These conditions of less alertness, less efficient cognitive processing, and less positive affect, may possibly then increase the likelihood of mind wandering or daydreaming. The finding of a moderation effect, in which the negative correlations between DF/MW and PA were weaker in morning-types than in evening-types or neutral-types, may also be related to social jet-lag, which is more of a problem for evening-types and neutral-types, than it is for morning-types.

### Limitations and Future Research

A limitation with this research is the reliance upon retrospective questionnaires. The ESS and PSQI have both shown weak consistency with more objective measures of sleep, but as Buysse et al. [65] note, this may partly be because these measures assess habitual patterns whereas objective measures like polysomnography take discrete measures; also, some aspects of sleep, like sleep quality, are subjective in nature [65]. Additionally, the scales used in this research generally showed good psychometric properties, replicated some previous findings, and showed some convergent validity. Also, there were some consistent findings from the three separate samples. Nevertheless, these findings should be replicated and extended. For example, future studies should include a wider age range. Older people seem to mind wander or daydream less [68], [69], but also generally (but not necessarily) tend to have more sleep problems [85], suggesting that the relationships between mind wandering, daydreaming and sleep quality may change over the lifespan. Also, the relationships between sleep, mind wandering and daydreaming could be explored in more detail in longitudinal research over an extended period of time, possibly using actigraphy for more objective measurement of sleep, in conjunction with thought-sampling to collect reports of mind wandering/daydreaming, and mood, while people do their usual routines (cf. [5], [25], [86], [87]). This should include data for weekdays and weekends, when sleep patterns typically differ [39]. Measures of other potential influences on the association between sleep quality and mind wandering also need to be taken. These may include drug or alcohol use [60], stressors, such as exams, and also stress about sleep itself. For instance, Alapine et al. [73] found that people who are more distressed by their poor sleep show more negative effects than do those who are less concerned; perhaps distress at poor sleep also modulates the frequency of mind wandering or daydreaming. There may also be genetic influences. Barclay et al. [66] report that chronotype is approximately 50% heritable, while sleep quality is approximately 40% heritable, and that substantially the same genes are implicated in both. Twin research on mind wandering or daydreaming tendencies and characteristics, and associations with sleep quality and chronotype, may be intriguing. Finally, an interesting possibility is that a sleep-related increase in daydreaming may to some extent be performing a compensatory/restorative function, similar to REM sleep, to make-up for lack of sleep, or REM deprivation [14].

### Conclusion

The present study has shown that the frequency of mind wandering or daydreaming is associated with various aspects of sleep quality. Although conclusions about causality cannot be made, the findings from this research, in conjunction with other studies, suggest a bi-directional relationship, in which some forms of mind wandering or daydreaming may be a cause and/or a consequence of poor sleep. These relationships are of practical significance, given that cognitive impairment related to sleep disruption brings increased risk of work-place and motor accidents [7]–[10], and is detrimental to learning and academic achievement [20], [63], [88]. Negatively valenced forms of mind wandering or daydreaming may be especially related to sleep quality: the relationships between negative affect, mind wandering and daydreaming, which have been the focus of much recent research and discussion (e.g., [15], [22], [25], [26], [27], [74], [82], [86], [87], [89]), were found to be partially mediated by poor sleep quality. Furthermore, the correlations between eveningness and mind wandering, and eveningness and daydreaming, were both jointly mediated by (low) positive affect and (poor) sleep quality, showing synchrony with evidence that sleep quality may also mediate the relationship between morningness and mindfulness [30]. Improving sleep quality may help to reduce the frequency of some forms of mind wandering or daydreaming, while reducing mind wandering, perhaps through mindfulness training [31], [32], may help to improve sleep quality. It seems that the relationships between aspects of sleep quality and forms of mind wandering or daydreaming may be a fruitful area for future research.

## References

[1] Klinger E (1999) Thought Flow: Properties and Mechanisms Underlying Shifts in Content. In: Singer JA, Salovey P editors. At Play in the Fields of Consciousness: Essays in Honour of Jerome L. Singer. Mahwah, New Jersey: LEA. 29–50.

[2] Smallwood J (2006) Schooler JW (2006) The Restless Mind. Psychol Bull 132(6): 946–948. 17073528 10.1037/0033-2909.132.6.946

[3] Singer JL (1966) Daydreaming: An introduction to the experimental study of inner experience. New York, NY: Random House.

[4] McVay JC, Kane MJ, Kwapil TR (2009) Tracking the train of thought from the laboratory into everyday life: An experience-sampling study of mind wandering across controlled and ecological contexts. Psychon B Rev 16(5): 857–863. 10.3758/PBR.16.5.857 PMC2760023 19815789

[5] Song X, Wang X (2012) Mind Wandering in Chinese Daily Lives – An Experience Sampling Study. PLoS ONE 7(9): e44423 10.1371/journal.pone.0044423 22957071 PMC3434139

[6] Mooneyham BW (2013) Schooler JW (2013) The Costs and Benefits of Mind-Wandering: A review. Can J Exp Psychol 67(1): 11–18. 23458547 10.1037/a0031569

[7] Durmer JS, Dinges DF (2005) Neurocognitive Consequences of Sleep Deprivation. Semin Neurol 25(1): 117–129. 15798944 10.1055/s-2005-867080

[8] Åkerstedt T (2007) Altered sleep/wake patterns and mental performance. Physiol Beh 90(2): 209–218. 10.1016/j.physbeh.2006.09.007 17049569

[9] Alhola P, Polo-Kantola P (2007) Sleep deprivation: Impact on cognitive performance. Neuropsychiatr Dis Treat 3(5) 553–567. PMC2656292 19300585

[10] Mallis MM, Banks S, Dinges JF (2008) Sleep and Circadian Control of Neurobehavioural Functions. In: Parasuraman R, Rizzo M, editors. Neuroergonomics: The Brain at Work. New York: Oxford University Press. 207–220.

[11] Braboszcz C, Delorme A (2011) Lost in thoughts: neural markers of low alertness during mind wandering. Neuroimage 54(4): 3040–3047. 20946963 10.1016/j.neuroimage.2010.10.008

[12] Antrobus JS, Singer JL, Greenberg S (1966) Studies in the stream of consciousness: experimental enhancement and suppression of spontaneous cognitive processes. Percept Motor Skill 23: 399–417.

[13] Kunzendorf R, Brown C, McGee D (1983) Hypnotizability: Correlations with daydreaming and sleeping. Psychol Rep 53(2): 406. 6647688 10.2466/pr0.1983.53.2.406

[14] Pritzl TJ (2003) The Effect of Experimentally Enhanced Daydreaming on an Electroencephalographic Measure of Sleepiness. Northern Carolina State University. Available: http://www.lib.ncsu.edu/resolver/1840.16/3833. Accessed 2013 Nov 30.

[15] Ottaviani C, Shapiro D, Couyoumdjian A (2013) Flexibility as the key for somatic health: From mind wandering to perseverative cognition. Biol Psychol 94: 38–43. 23680439 10.1016/j.biopsycho.2013.05.003

[16] Mikulincer M, Babkoff H, Caspy T, Weiss H (1990) The impact of cognitive interference on performance during prolonged sleep loss. Psychol Res 52(1): 80–86. 2377728 10.1007/BF00867216

[17] Harvey AG, Payne S (2002) The management of unwanted pre-sleep thoughts in insomnia: distraction with imagery versus general distraction. Behav Res Ther 40(3): 267–277. 11863237 10.1016/s0005-7967(01)00012-2

[18] Zoccola PM, Dickerson SS, Lam S (2009) Rumination predicts longer sleep onset latency after an acute psychosocial stressor. Psychosom Med 71(7): 771–775. 19622710 10.1097/PSY.0b013e3181ae58e8

[19] Lund HG, Reider BD, Whiting AB, Prichard JR (2010) Sleep patterns and predictors of disturbed sleep in a large population of college students. J Adolescent Health 46(2): 124–132. 10.1016/j.jadohealth.2009.06.016 20113918

[20] Chung KF, Cheung MM (2008) Sleep-wake patterns and sleep disturbance among Hong Kong Chinese adolescents. Sleep 31(2): 185–194. 18274265 10.1093/sleep/31.2.185 PMC2225574

[21] Verlander LA, Benedict JO, Hanson DP (1999) Stress and sleep patterns of college students. Percept Motor Skill 88(3): 893–898. 10.2466/pms.1999.88.3.893 10407896

[22] Ottaviani C, Couyoumdjian A (2013) Pros and cons of a wandering mind: a prospective study. Front Psychol 4: 524 10.3389/fpsyg.2013.00524 23966964 PMC3743222

[23] Giambra LM, Traynor TD (1978) Depression and daydreaming: An analysis based on self-ratings. J Clin Psycho 34(1): 14–25. 641165

[24] Smallwood J, O’Connor RC, Sudbery MV, Obonsawin M (2007) Mind-wandering and dysphoria. Cognition Emotion 21(4): 816–842.

[25] Killingsworth MA, Gilbert DT (2010) A Wandering Mind is an Unhappy Mind. Science 330: 932. 21071660 10.1126/science.1192439

[26] Smallwood J, Fitzgerald A, Miles LK, Phillips LH (2009) Shifting Moods, Wandering Minds: Negative Moods Lead the Mind to Wander. Emotion 9(2): 271–276. 19348539 10.1037/a0014855

[27] Stawarczyk D, Majerus S, Van der Linden M, D’Argembeau A (2012) Using the daydreaming frequency scale to investigate the relationships between mind-wandering, psychological well-being, and present-moment awareness. Front Psychol 3: 363 10.3389/fpsyg.2012.00363 23055995 PMC3457083

[28] Grossman P, Van Dam NT (2011) Mindfulness, by any other name…: trials and tribulations of sati in western psychology and science. Contemporary Buddhism 12(1): 219–239.

[29] Brown KW, Ryan RM (2003) The Benefits of Being Present: Mindfulness and Its Role in Psychological Well-Being. J Pers Soc Psychol. 84(4): 822–848. 10.1037/0022-3514.84.4.822 12703651

[30] Howell AJ, Digdon NL, Buro K, Sheptycki AR (2008) Relations among mindfulness, well-being and sleep. Pers Indiv Differ 45(8): 773–777.

[31] Ong JC, Shapiro SL, Manber R (2009) Mindfulness meditation and cognitive behavioral therapy for insomnia: a naturalistic 12-month follow-up. Explore 5(1): 30–36. 19114261 10.1016/j.explore.2008.10.004 PMC4766838

[32] Mrazek MD, Smallwood J (2012) Schooler JW (2012) Mindfulness and Mind-Wandering: Finding Convergence Through Opposing Constructs. Emotion 12(3): 442–448. 22309719 10.1037/a0026678

[33] Carciofo R, Du F, Song N, Zhang K (2013) Chronotype and time-of-day correlates of mind wandering and related phenomena. Biol Rhythm Res doi:10.1080/09291016.2013.790651.

[34] Johnson EO, Roth T, Breslau N (2006) The association of insomnia with anxiety disorders and depression: Exploration of the direction of risk. J Psychiatr Res 40(8): 700–708. 16978649 10.1016/j.jpsychires.2006.07.008

[35] Riemann D, Spiegelhalder K, Feige B, Voderholzer U, Berger M, et al (2010) The hyperarousal model of insomnia: A review of the concept and its evidence. Sleep Med Rev 14(1): 19–31. 19481481 10.1016/j.smrv.2009.04.002

[36] Bixler EO, Vgontzas AN, Lin HM, Calhoun SL, Vela-Bueno A, et al (2005) Excessive daytime sleepiness in a general population sample: The role of sleep apnea, age, obesity, diabetes, and depression. J Clin Endocr Meta 90(8): 4510–4515. 10.1210/jc.2005-0035 15941867

[37] Horne JA, Östberg O (1976) A self-assessment questionnaire to determine morningness-eveningness in human circadian rhythms. Int J Chronobiol 4: 97–110. 1027738

[38] Adan A, Archer SN, Hidalgo MP, Di Milla L, Natale V, et al (2012) Circadian Typology: A Comprehensive Review. Chronobiol Int 29(9): 1153–1175. 23004349 10.3109/07420528.2012.719971

[39] Taillard J, Philip P, Bioulac B (1999) Morningness/eveningness and the need for sleep. J Sleep Res 8(4): 291–295. 10646169 10.1046/j.1365-2869.1999.00176.x

[40] Sukegawa M, Noda A, Morishita Y, Ochi H, Miyata S, et al (2009) Sleep and lifestyle habits in morning and evening types of human circadian rhythm. Biol Rhythm Res 40(2): 121–127.

[41] Giannotti F, Cortesi F, Sebastiani T, Ottaviano S (2002) Circadian preference, sleep and daytime behaviour in adolescence. J Sleep Res 11(3): 191–199. 12220314 10.1046/j.1365-2869.2002.00302.x

[42] Vardar E, Vardar SA, Molla T, Kaynak C, Ersoz E (2008) Psychological symptoms and sleep quality in young subjects with different circadian preferences. Biol Rhythm Res 39(6): 493–500.

[43] Fernández-Mendoza J, Ilioudi C, Montes MI, Olavarrieta-Bernardino S, Aguirre-Berrocal A, et al (2010) Circadian preference, nighttime sleep and daytime functioning in young adulthood. Sleep Biol Rhythms 8(1): 52–62.

[44] Wittmann M, Dinich J, Merrow M, Roenneberg T (2006) Social jetlag: misalignment of biological and social time. Chronobiol Int 23(1–2): 497–509. 16687322 10.1080/07420520500545979

[45] Biss RK, Hasher L (2012) Happy as a Lark: Morning-Type Younger and Older Adults Are Higher in Positive Affect. Emotion 12(3): 437–441. 22309732 10.1037/a0027071 PMC3399900

[46] Schooler JW, Smallwood J, Christoff K, Handy TC, Reichle ED, et al (2011) Meta-awareness, perceptual decoupling and the wandering mind. Trends Cogn Sci 15(7): 319–326. 21684189 10.1016/j.tics.2011.05.006

[47] Mrazek MD, Phillips DT, Franklin MS, Broadway JM, Schooler JW (2013) Young and restless: validation of the Mind-Wandering Questionnaire (MWQ) reveals disruptive impact of mind-wandering for youth. Front Psychol 4. doi:10.3389/fpsyg.2013.00560. 10.3389/fpsyg.2013.00560 PMC3753539 23986739

[48] Adan A, Almirall H (1991) Horne & Östberg Morningness-Eveningness Questionnaire: A Reduced Scale. Pers Indiv Differ 12(3): 241–253.

[49] Carciofo R, Du F, Song N, Qi Y, Zhang K (2012) Age-related chronotype differences in Chinese, and reliability assessment of a reduced version of the Chinese Morningness-Eveningness Questionnaire. Sleep Biol Rhythms 10: 310–318.

[50] Li SX, Li QQ, Wang XF, Liu LJ, Liu Y, et al (2011) Preliminary test for the Chinese version of the Morningness–Eveningness Questionnaire. Sleep Biol Rhythms 9: 19–23.

[51] Singer JL, Antrobus JS (1972) Daydreaming, Imaginal Processes, and Personality: A Normative Study. In: PW Sheehan editor. The Nature and Function of Imagery. New York: Academic Press. 175–202. IPI scales available at: http://themeasurementgroup.com/evaluationtools/ipi.htm. Accessed 2012 April 4.

[52] Buysse DJ, Reynolds III CF, Monk TH, Berman SR, Kupfer DJ (1989) The Pittsburgh Sleep Quality Index: a new instrument for psychiatric practice and research. Psychiat Res 28(2): 193–213. 10.1016/0165-1781(89)90047-4 2748771

[53] Liu X, Tang M, Hu L (1996) Reliability and Validity of the Pittsburgh Sleep Quality Index. Chinese Journal of Psychiatry 29(2): 103–107. [Chinese language].

[54] Johns MW (1991) A new method for measuring daytime sleepiness: The Epworth sleepiness scale. Sleep 14(6): 540–545. 1798888 10.1093/sleep/14.6.540

[55] Peng LL, Li JR, Sun JJ, Li WY, Sun YM, et al.. (2011) Reliability and validity of the simplified Chinese version of Epworth sleepiness scale. Chin J Otorhinolaryngol Head and Neck Surg 46(1): 44–49. [Chinese language]. 21429336

[56] Watson D, Clark LA, Tellegen A (1988) Development and validation of brief measures of positive and negative affect: the PANAS scales. J Pers Soc Psychol 54(6): 1063–1070. 3397865 10.1037//0022-3514.54.6.1063

[57] Huang L, Yang T, Ji Z (2003) Applicability of the positive and negative affect scale in Chinese. Chinese Mental Health Journal 17(1): 54–56. [Chinese language].

[58] Preacher KJ, Hayes AF (2004) SPSS and SAS procedures for estimating indirect effects in simple mediation models. Behav Res Meth Ins C 36(4): 717–731. 10.3758/bf03206553 15641418

[59] Preacher KJ, Hayes AF (2008) Asymptotic and resampling strategies for assessing and comparing indirect effects in multiple mediator models. Behav Res Methods 40(3): 879–891. 18697684 10.3758/brm.40.3.879

[60] Kenney SR, LaBrie JW, Hummer JF, Pham AT (2012) Global sleep quality as a moderator of alcohol consumption and consequences in college students. Addict Behav 37(4): 507–512. 22285119 10.1016/j.addbeh.2012.01.006 PMC4329778

[61] Norlander T, Johansson Å, Bood SÅ (2005) The affective personality: Its relation to quality of sleep, well-being and stress. Soc Beh Personal 33(7): 709–722.

[62] Cheng SH, Shih CC, Lee IH, Hou YW, Chen KC, et al (2012) A study on the sleep quality of incoming university students. Psychiat Res 197(3): 270–274. 10.1016/j.psychres.2011.08.011 22342120

[63] Suen LK, Hon KL, Tam WW (2008) Association between sleep behavior and sleep-related factors among university students in Hong Kong. Chronobiol Int 25(5): 760–775. 18780202 10.1080/07420520802397186

[64] Johns MW (2000) Sensitivity and specificity of the multiple sleep latency test (MSLT), the maintenance of wakefulness test and the Epworth sleepiness scale: failure of the MSLT as a gold standard. J Sleep Res 9(1): 5–11. 10733683 10.1046/j.1365-2869.2000.00177.x

[65] Buysse DJ, Hall ML, Strollo PJ, Kamarck TW, Owens J, et al (2008) Relationships between the Pittsburgh Sleep Quality Index (PSQI), Epworth Sleepiness Scale (ESS), and clinical/polysomnographic measures in a community sample. J Clin Sleep Med 4(6): 563–571. 19110886 PMC2603534

[66] Barclay NL, Eley TC, Buysse DJ, Archer SN, Gregory AM (2010) Diurnal preference and sleep quality: same genes? A study of young adult twins. Chronobiol Int 27(2): 278–296. 20370470 10.3109/07420521003663801

[67] Selvi Y, Aydin A, Gulec M, Boysan M, Besiroglu L, et al (2012) Comparison of dream anxiety and subjective sleep quality between chronotypes. Sleep Biol Rhythms 10(1): 14–22.

[68] Giambra LM (1974) Daydreaming across the lifespan: late adolescence to senior citizen. Aging and Hum Dev 5(2): 115–140. 10.2190/7AEJ-T3MA-QLGD-CCF5 4430512

[69] Giambra LM (1989) Task-Unrelated-Thought Frequency as a Function of Age: A Laboratory Study. Psychol Aging 4(2): 136–143. 2789741 10.1037/0882-7974.4.2.136

[70] Harvey AG, Tang NKY, Browning L (2005) Cognitive approaches to insomnia. Clin Psychol Rev 25(5): 593–611. 15979771 10.1016/j.cpr.2005.04.005

[71] Klinger E (1978) Modes of Normal Conscious Flow. In: Pope KS, Singer JL, editors. The Stream of Consciousness. New York: Plenum Press. 225–258.

[72] Killgore WDS, Schwab ZJ, Weiner MR (2012) Self-reported nocturnal sleep duration is associated with next-day resting state functional connectivity. NeuroReport 23(13): 741–745. 22872066 10.1097/WNR.0b013e3283565056

[73] Alapin I, Fichten CS, Libman E, Creti L, Bailes S, et al (2000) How is good and poor sleep in older adults and college students related to daytime sleepiness, fatigue, and ability to concentrate? J Psychosom Res 49(5): 381–390. 11164064 10.1016/s0022-3999(00)00194-x

[74] Mason MF, Brown K, Mar RA, Smallwood J (2013) Driver of discontent or escape vehicle: the affective consequences of mindwandering. Front Psychol4: 477 10.3389/fpsyg.2013.00477 PMC3722495 23898317

[75] Smallwood J, Andrews-Hanna J (2013) Not all minds that wander are lost: the importance of a balanced perspective on the mind-wandering state. Front Psychol4: 441 10.3389/fpsyg.2013.00441 PMC3744871 23966961

[76] McVay JC, Kane MJ (2010) Does mind wandering reflect executive function or executive failure? Comment on Smallwood and Schooler (2006) and Watkins (2008). Psychol Bull 136: 188–197. 20192557 10.1037/a0018298 PMC2850105

[77] Sayette MA, Reichle ED (2009) Schooler JW (2009) Lost in the Sauce: The Effects of Alcohol on Mind Wandering? Psychol Sci 20(6): 747–752. 19422627 10.1111/j.1467-9280.2009.02351.x PMC2724753

[78] Smallwood J (2010) Why the Global Availability of Mind Wandering Necessitates Resource Competition: Reply to McVay and Kane. Psychol Bull. 136(2): 202–207.

[79] Levinson DB, Smallwood J, Davidson RJ (2012) The Persistence of Thought: Evidence for a Role of Working Memory in the Maintenance of Task-Unrelated Thinking. Psychol Sci 23(4): 375–380. 22421205 10.1177/0956797611431465 PMC3328662

[80] Smallwood J (2013) Distinguishing How From Why the Mind Wanders: A Process–Occurrence Framework for Self-Generated Mental Activity. Psychol Bull 139(3): 519–535. 23607430 10.1037/a0030010

[81] Mar RA, Mason MF, Litvack A (2012) How daydreaming relates to life satisfaction, loneliness, and social support: The importance of gender and daydream content. Conscious Cogn 21(1): 401–407. 22033437 10.1016/j.concog.2011.08.001

[82] McMillan R, Kaufman SB, Singer JL (2013) Ode to Positive Constructive Daydreaming. Front Psychol 4: 626 10.3389/fpsyg.2013.00626 24065936 PMC3779797

[83] Singer JL (1978) Experimental Studies of Daydreaming and the Stream of Thought. In: Pope KS, Singer JL editors. The Stream of Consciousness. New York: Plenum Press. 187–223.

[84] Singer JL, Antrobus JS (1963) A factor-analytic study of daydreaming and conceptually-related cognitive and personality variables. Percept Motor Skill 17: 187–209. 10.2466/pms.1963.17.1.187 14045737

[85] Vitiello MV (2009) Recent Advances in Understanding Sleep and Sleep Disturbances in Older Adults Growing Older Does Not Mean Sleeping Poorly. Curr Dir Psychol Sci 18(6): 316–320.

[86] Franklin MS, Mrazek MD, Anderson CL, Smallwood J, Kingstone A, et al (2013) The silver lining of a mind in the clouds:interesting musings are associated with positive mood while mind-wandering. Front Psychol 4: 583 10.3389/fpsyg.2013.00583 24009599 PMC3755259

[87] Poerio GL, Totterdell P, Miles E (2013) Mind-wandering and negative mood: Does one thing really lead to another? Conscious Cogn 22(4): 1412–1421. 24149091 10.1016/j.concog.2013.09.012

[88] Curcio G, Ferrara M, De Gennaro L (2006) Sleep loss, learning capacity and academic performance. Sleep Med Rev 10(5): 323–337. 16564189 10.1016/j.smrv.2005.11.001

[89] Marchetti I, Koster EH, De Raedt R (2012) Mindwandering heightens the accessibility of negative relative to positive thought. Conscious Cogn 21(3): 1517–1525. 22726693 10.1016/j.concog.2012.05.013

